# Does improved functional performance help to reduce urinary incontinence in institutionalized older women? a multicenter randomized clinical trial

**DOI:** 10.1186/1471-2318-12-51

**Published:** 2012-09-06

**Authors:** Erwin CPM Tak, Ariëtte van Hespen, Paula van Dommelen, Marijke Hopman-Rock

**Affiliations:** 1 TNO Expertise center Life Style, P.O. Box 2215, Leiden, 2301 CE, The Netherlands; 2 Body@Work, Research Center Physical Activity, Work and Health, TNO-VU, University Medical Center, Amsterdam, The Netherlands; 3 Department of Public and Occupational Health/EMGO Institute for Health and Care Research (EMGO+), VU University Medical Center, Amsterdam, The Netherlands

## Abstract

**Background:**

Urinary incontinence (UI) is a major problem in older women. Management is usually restricted to dealing with the consequences instead of treating underlying causes such as bladder dysfunction or reduced mobility.

The aim of this multicenter randomized controlled trial was to compare a group-based behavioral exercise program to prevent or reduce UI, with usual care. The exercise program aimed to improve functional performance of pelvic floor muscle (PFM), bladder and physical performance of women living in homes for the elderly.

**Methods:**

Twenty participating Dutch homes were matched and randomized into intervention or control homes using a random number generator. Homes recruited 6–10 older women, with or without UI, with sufficient cognitive and physical function to participate in the program comprising behavioral aspects of continence and physical exercises to improve PFM, bladder and physical performance. The program consisted of a weekly group training session and homework exercises and ran for 6 months during which time the control group participants received care as usual. Primary outcome measures after 6 months were presence or absence of UI, frequency of episodes (measured by participants and caregivers (not blinded) using a 3-day bladder diary) and the Physical Performance Test (blinded). Linear and logistic regression analysis based on the Intention to Treat (ITT) principle using an imputed data set and per protocol analysis including all participants who completed the study and intervention (minimal attendance of 14 sessions).

**Results:**

102 participants were allocated to the program and 90 to care as usual. ITT analysis (n = 85 intervention, n = 70 control) showed improvement of physical performance (intervention +8%; control −7%) and no differences on other primary and secondary outcome measures. Per protocol analysis (n = 51 intervention, n = 60 control) showed a reduction of participants with UI (intervention −40%; control −28%) and in frequency of episodes (intervention −51%; control −42%) in both groups; improvement of physical performance (intervention + 13%; control −4%) was related to participation in the exercise program.

**Conclusions:**

This study shows that improving physical performance is feasible in institutionalized older women by exercise. Observed reductions in UI were not related to the intervention. [Current Controlled Trials ISRCTN63368283]

## Background

Urinary incontinence (UI) is one of the major problems in the geriatric population with high impact on quality of life in the community-dwelling [1] and the institutionalized older adults [2]. High prevalence [3,4] and incidence rates are reported [5], especially among older women in long-term care facilities with prevalence running between 50 to 90% [6,7].

Although conservative treatment for UI, such as pelvic floor muscle training (PFMT) and bladder training (BT), have been proven effective in both adult women [8-10], and community-dwelling older adults [11,12], current practice for institutionalized women focuses on managing the consequences by providing incontinence pads and toileting assistance [7,13,14] instead of treating underlying conditions or causes of UI.

Functional decline of cognition and mobility are among the most important independent risk factors for UI in community-dwelling women [15-17], and in older women living in institutions [6]. A decline in mobility may even lead to UI with no urogenital pathology, therefore addressing functional decline in order to prevent or reduce UI in older adults seems to be a promising strategy [18].

Strategies such as prompted voiding and individual physical training have been shown to have a positive effect on frail nursing home residents. These interventions significantly reduce the frequency of incontinence episodes and improve mobility endurance [19,20]even in people with mental and physical impairments [2,12,21,22]. The main disadvantages of these interventions are the increased workload for nursing staff and high costs which could obstruct large-scale implementation. Recent reviews report that there is still a lack of prevention studies on maintaining continence in care homes and that factors associated with incontinence need to be considered [23,24].

For these reasons it would be worthwhile to develop a strategy that targets the main causes and risk factors of UI without increasing the workload for nursing staff in long-term care facilities.

To evaluate such a strategy we developed a group-based physical exercise program to be delivered by physical therapists. It consisted of exercises that target the functional causes of UI both directly by strengthening of the pelvic floor muscles and bladder training, and indirectly by improving physical performance relevant to continence behavior. The aim of this study was to evaluate the effectiveness of this group-based program by improving functional performance in institutionalized older women. We hypothesized that on comparing a group of participants in the exercise program with those receiving usual care, the number of participants with UI and the frequency of UI episodes would be reduced and physical performance relevant to continence behavior (mobility, dexterity etc.) would be improved.

## Methods

In designing the trial and developing the intervention, results from an epidemiological study into urinary incontinence in homes for the elderly were used [7]. One finding was that despite the high prevalence of UI in homes for the elderly, there was still a taboo among residents on talking about these problems or addressing them in public. For this reason, the intervention was designed to also include women without UI, thereby avoiding the stigma of UI when participating. The goal of the intervention for the participants were to prevent development of UI and to improve physical performance. To avoid contamination between the intervention and the control groups, it was decided to allow only one intervention or one control group per home, and randomize on the level of the home.

The study was approved by the TNO medical ethics committee. This report has been drafted in accordance with the CONSORT criteria [25] and adds to a previously published Dutch report of this study which included the per-protocol analysis [26] and is now elaborated in this report with an Intention to Treat analysis on imputed data focusing on functional performance.

Power calculations revealed that to be able to show a 20% overall reduction in UI, 103 participants per group (power 0.8; alpha = 0.05) were needed. A multilevel design with the same number of participants, 10 homes per group and an Intra Class Correlation (ICC) of 0.05 would make a reduction of 23% detectable. We aimed to recruit 103 participants per group, with 7 extra per group to adjust for withdrawal.

### Design and procedure

A multicenter, randomized controlled trial was carried out to evaluate the exercise program ‘Incondition’. Homes for the elderly were recruited via a newsletter distributed by the National Organization for Institutionalized Care in the Netherlands and through direct mailing. In total, 27 homes were interested 20 of which finally agreed to participate. Homes that withdrew feared that in cooperating with the study the workload for staff members would be too large. Participating homes were matched and randomized into either the intervention condition (Incondition program) or the control condition (care as usual). Matching was done to assure that intervention and control homes were comparable by using the following factors: prevalence of UI and use of incontinence pads, number of residents, percentage of residents receiving psycho geriatric care (i.e. for dementia), number of staff members, percentage of residents with impaired mobility, and average level of care per participant in minutes. These data were provided by the participating homes. Matched pairs were randomized using a random number generator. Comparing participating homes using these factors showed that intervention and control homes were comparable, with the exception of the percentage of use of incontinence pads which was slightly higher in the control homes (100% vs. 89% of the homes).

Before randomization, participating homes were asked to recruit between 6–10 residents. Homes that recruited less than 6 residents were excluded. Inclusion criteria for participants were being female and having sufficient cognitive and physical function to allow them to participate in the program. Residents who were catheterized were excluded. Inclusion criteria were initially evaluated by staff and at baseline measurement checked with validated instruments. Cognitive function was measured with the Cognitive Screening Test (CST) [27]; participants with a score of 9.6 or lower were excluded. The Barthel Index [28] was used to exclude those who could not use the toilet independently. Eligible residents were contacted by staff members and given written and oral information on the study. All residents who agreed to participate filled out an informed consent form before taking part.

Measurements were taken at baseline, halfway through (3 months), and at the end of the intervention period (6 months). Measurements, including an interview and physical tests, were carried out by students trained in physical therapy (blinded). For additional self-report measures (i.e. bladder diary), staff from the participating homes (not blinded) assisted participants (not blinded).

### Intervention

The intervention was based on the results of a previous inventory in Dutch homes for the elderly [7]. It addressed the main causes of UI in this population: strength of pelvic floor muscles, bladder control and mobility. It had to be accessible, fun, easy to understand, of low intensity, and interesting for women both with and without UI. The Incondition program consisted of weekly, 1-hour training sessions for groups of 6–10 women over a period of 22 weeks.

Each session consisted of behavioral instructions and physical exercises. The behavioral element aimed to improve the control of micturition by improving knowledge about continence, improving toilet behavior (position, relaxation etc.), BT, and PFMT including relaxation and breathing.

The aim of the physical exercises was to increase the functional ability to use the toilet independently and in time. Exercises were kept functional and pleasant, and used materials to enhance compliance. The 30-minute exercise session included warming up, exercises to improve the mobility of the upper extremities, hand function, standing up and sitting down on a chair or bed, walking, and cooling down.

Participants received a written leaflet containing guidelines on good toilet behavior and micturition. After each session, homework exercises were given, if possible on an individual basis, and evaluated at the start of the following session. The intervention was delivered by physical therapists specialized in PFMT who had experience with group training and affinity with the elderly. The physical therapists received special training to carry out the intervention correctly.

At the end of the program, data on compliance, reasons for missing sessions and for dropping out, general opinion of the program, subjective improvement, and adverse effects of the program were collected.

The participants in the control group received care as usual, including prescription of incontinence pads (100% of participants with UI) and toilet assistance.

### Measurements/instruments

Primary outcome measures were UI status, severity of UI and physical performance. Physical performance was measured with the Physical Performance Test (PPT) which assesses multiple domains of physical function by timing how long it takes to perform tasks of varying degrees of difficulty (i.e. writing a sentence, putting on and taking off a jacket, turning around 360 degrees, walking 15 meters) [29]. Total score ranges from 0 (worst) to 28. Next, involuntary urine loss was measured using a 3-day bladder diary to evaluate the presence and severity (i.e. frequency of episodes) of urinary loss. Participants, assisted by their caregivers, recorded micturition and fluid intake (the latter only at three months). For each participant UI status was defined in two ways. First UI status was defined as having at least one episode of involuntary urine loss during this 3-day period, resulting in yes or no UI. Secondly, UI frequency was defined as the total number of episodes during 3 days.

Secondary outcome measures included quality of life measured with two self-report questionnaires: the SF-12 questionnaire which describes the mental and physical health status of adults [30], score ranges from 0 (worst) to 100, and the Incontinence Quality of Life Instrument (I-QOL) [31] which contains 22 items that measure incontinence-related quality of life, with a total score ranging from 0 (worst) to 110.

At baseline, descriptive statistics included information on age (years), level of education (low, intermediate, high), length of stay (months), physical disability, cognition, subjective symptoms of urinary and fecal incontinence, use of incontinence pads, and comorbidity (number of chronic diseases). The level of physical disability was assessed with the Barthel Index [28], which consists of 10 items concerning basic activities of daily living (ADL). Scores range from 0 to 20, with a higher score indicating greater independence.

Cognition was evaluated with the 14-item version of the Cognitive Screening Test [27]. The maximum score of 14 indicates normal cognitive function. All other measures were part of the interview.

### Analysis

Two separate analyses were done to evaluate the effect of the program: 1) Intention to Treat (ITT) analysis performed in all participants who started the Incondition program, or care as usual, and 2) a per protocol analysis of the data of all participants who completed the study and for the intervention group those who had attended a minimum of 14 sessions. For the ITT analysis, linear and logistic regression analyses were performed using the baseline measure as confounder. We restricted the use of mixed-effects multilevel analyses to those cases of significant differences between intervention and control groups because the imputation program used to enhance the data set did not take the multilevel structure of our data into consideration. We used Multivariate Imputation by Chained Equations (MICE) R version 2.12.2 [32]. The main reason for imputation was that caregivers were not always able to assist participants with their bladder diaries, some of which were not completed properly. In addition, participants were not always able to comply due to illness or absence. The imputation model included all outcome measures and characteristics shown in Table 1 and Table 2, as well as the number of the home. In total, five predictions were conducted and then pooled to produce estimates and confidence intervals that incorporated missing-data uncertainty. P-values < 0.05 (two-sided) were considered statistically significant.

**Table 1:** Baseline characteristics of intervention group and control group

	**Intervention group (n = 85)**	**Control group (n = 70)**
Age, years (SD)	84.6 (6.5)	84.7 (5.7)
Education(N)		
High (%)	14	6
Intermediate (%)	19	20
Low (%)	52	44
Cognition, 0-14‡ (SD)	12.7 (2.0)	12.7 (1.4)
Barthel index, 0-20† (SD)	16.1 (3.6)	15.4 (2.8)
Length of Stay, months (SD)	61.6 (65.5)	42.1 (43.6)
Subjective urinary incontinence (# participants)	46	39
Subjective fecal incontinence (# participants)	31	20
Comorbidity, number of chronic illnesses (SD)	1.8 (1.9)	3.0 (2.3)***
Number of incontinence pads in 3 weeks (SD)	63.7 (43.6)	54.4 (31.2)

*** p < .001; ‡ higher score indicates better performance; † higher sco1re indicates worse performance.

**Table 2:** Regression analysis for intervention (n = 85) and control (n = 70) group

	**Baseline T1**	**3 months T2**	**6 months T3**	***Test-statistic (T2-T1, T3-T1)***
Number of participants with UI				
Intervention group	40	37	40	0.66 (0.15,2.90), 0.80 (0.34,1.88)
Control group	40	38	34	
Frequency incontinence episodes (Number/ 3 days); mean (sd)				
Intervention group	8.0 (11.0)	6.6 (9.1)	9.0 (11.4)	−1.71 (−5.99,2.56), 1.38 (−3.03,5.79)
Control group	9.5 (11.5)	7.4 (10.1)	7.1 (9.6)	
Physical Performance Test (0-28†); mean (sd)				
Intervention group	17.2 (4.87)		18.5 (4 14)	3.21 (1.81,4.62), p < 0.001
Control group	15.8 (5.16)		14.7 (4.27)	
Health Related Quality of Life SF-12 (mental; 0-100); mean (sd)				
Intervention group	47.0 (14.2)		52.0 (10.0)	0.07 (−4.24,4.39)
Control group	46.0 (12.8)		51.3 (8.5)	
Health Related Quality of Life SF-12 (physical; 0-100†); mean (sd)				
Intervention group	34.7 (12.0)		38.3 (11.6)	1.70 (−3.39,6.80)
Control group	34.1 (10.1)		35.0 (12.1)	
Specific Quality of Life (I-QOL; 0-110†); mean (sd)				
Intervention group	68.9 (17.9)		65.7 (15.6)	−3.30 (−10.2,3.63)
Control group	62.2 (17.7)		66.2 (15.6)	

*corrected for chronic status.

† Higher score indicates better performance.

## Results

After randomization, two of the control homes dropped out because they were not able to recruit the minimum of 6 participating residents. The remaining 18 homes enrolled a total of 192 participants, of which 22 withdrew before baseline measurement and 15 were not eligible.

Baseline characteristics (Table 1), showed the average age of participants to be around 85 years, most having a lower level of education and an average stay in a home of 42–61 months. Slightly more then half of the study participants (54%) indicated they suffered from UI. They used an average of 54 to 64 incontinence pads every three weeks.

**Figure 1:**
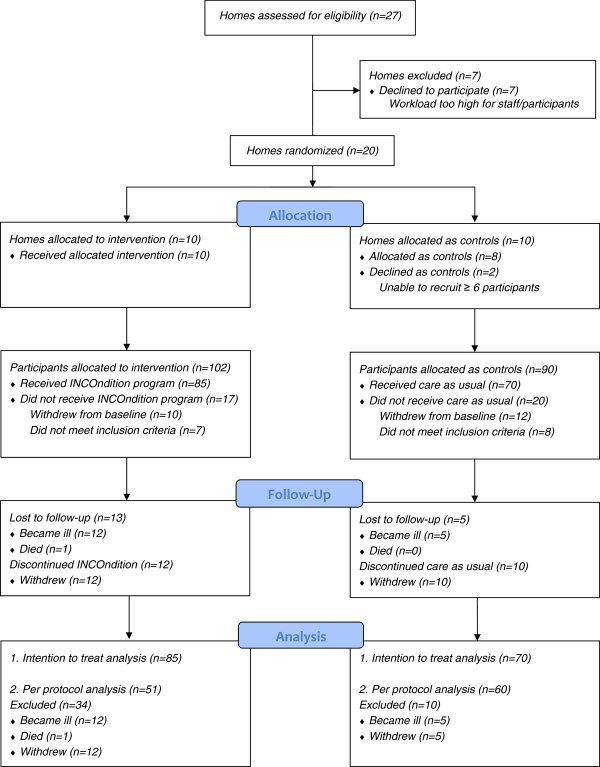
Study flowchart.

During the study period, 18 participants were lost to follow-up and 22 dropped out for various reasons (see Figure 1). Lack of motivation and not being satisfied with the program or physical therapist were reasons for dropping out of the program (n = 12). Of the participants who completed the study, 40% attended all training sessions, 30% missed one session, and 30% missed two or more sessions. Illness and other appointments were reasons for missing sessions. More than 50% of the participants indicated they did their homework exercises, 11% did only the pelvic floor exercises, and 3% only the physical exercises; 25% did not do the exercises independently. Of the participants who did their homework exercises, 33% said they did them more than once a day, 25% once a day, and 20% more than twice a week. About 65% of the participants indicated that they would certainly keep doing these exercises after the program had ended.

The linear and logistic regression analysis performed over all participants that started the Incondition program or care as usual (n = 155) showed, with the exception of physical performance, no differences in primary and secondary outcome measures (Table 2). The number of participants with UI declined slightly in both the intervention and control groups, while the frequency of incontinence episodes increased slightly in the intervention group after a decline at three months compared with a steady decline in the control group. UI-related quality of life seemed to improve more in the control group, but if we adjust for the multilevel structure this was not statistically significant. Physical performance (PPT) significantly improved in the intervention group (+8%) compared to a decline in the control group (−7%).

In Table 3 the per protocol analysis shows a decline of almost 20% of participants with UI (as registered with the bladder diary) at 3 months, and 40% at 6 months for those participating in the Incondition program. In the control group however, there was also a decline of 13% and 28% respectively. There was no significant difference between the two groups.

**Table 3:** Results Per Protocol Analysis

	**Baseline T1**	**3 months T2**	**6 months T3**	***Test-statistic***
Number of participants with UI				
Intervention group	29/51	25/51	18/51	Z = −.053‡
Control group	36/60	31/60	26/60	Z = −.531§
Frequency incontinence episodes (Number/ 3 days); mean (sd)				
Intervention group (n = 51)	9.0 (12.2)	6.6 (9.6)	4.4 (7.4)	F = 0.1
Control group (n = 60)	9.3 (11.3)	7.1 (10.5)	5.4 (8.5)	
Physical Performance Test (0-28†); mean (sd)				
Intervention group (n = 51)	16.7 (4.7)	-	18.8 (3.9)	F = 10.1*
Control group (n = 60)	16.4 (4.5)	-	15.7 (4.5)	
Health Related Quality of Life SF-12 (mental; 0-100†); mean (sd)				
Intervention group (n = 51)	51.4 (10.5)	-	49.9 (10.9)	F = 1.5
Control group (n = 60)	52.7 (10.1)	-	53.6 (7.7)	
Health Related Quality of Life SF-12 (physical; 0-100†); mean (sd)				
Intervention group (n = 51)	35.5 (11.0)	-	36.6 (11.2)	F = 2.4
Control group (n = 60)	34.7 (10.2)	-	32.4 (11.2)	
Specific Quality of Life (I-QOL; 0-110†); mean (sd)				
Intervention group (n = 51)	66.6 (15.0)	-	63.5 (15.0)	F = 0.5∥
Control group (n = 60)	59.1 (15.2)	-	66.1 (13.2)	

* p < .01.

† Higher score indicates better performance.

‡ difference between baseline and 3 months (Mann–Whitney Test).

§ difference between baseline and 6 months (Mann–Whitney Test).

∥Corrected for baseline difference.

The frequency of incontinence episodes decreased by 27% at 3 months and by 51% at 6 months in the intervention group, and by 24% and 42%, respectively, in the control group. This did not prove to be significantly different between the two groups.

Physical performance (PPT) was significantly better in the intervention group (improved by 13%) than in the control group (− 4%). There was no difference in quality of life between the two groups. The SF-12 scores for mental and physical functioning showed no differences over time or between groups. The seemingly better scores of the control group on the I-QOL were no longer found after correction for baseline differences.

More than 80% of the participants who completed the program considered it to be good to very good. Fourteen percent said the program was neither good nor bad, and 3% said it was bad to very bad. When asked if they had benefited from the program, 55% said they had benefited a lot, 11% a little, 15% were neutral, and 20% indicated they had not benefited. Almost 40% said they were better able to postpone or control micturition, 33% thought they had more information, 9% was especially satisfied with the contact with others, 7% reached the toilet quicker, and 5% indicated less loss of urine.

### Adverse effects

Ten percent of the participants in the program complained of muscle pain, fatigue, trouble with breathing or more involuntary urine loss.

## Discussion

The exercise program ‘Incondition’ was developed to prevent or reduce urinary incontinence in older women in homes for the elderly and to improve physical performance. Participation in the intervention resulted in a small improved physical performance but a reduction in UI was reported only in compliant study participants and irrespective of group allocation.

Being able to improve physical performance is important since the inability to walk or transfer independently is associated with UI in frail older individuals [15,33]. The improved physical performance in the intervention group did not lead to a reduction in UI. It might be that the improvement was too small for a change in UI or there were other more dominant causes that were not improved by the intervention. The exercise program itself was well received by most participants, considered satisfactory, and contributed to subjective improvement.

Interestingly enough these results show that reduction in UI in frail elderly women in homes for the elderly is possible, even without participating in an exercise program aimed at reducing the major causes of UI in this group. A possible explanation for the improvement in the control group could be that the close monitoring of incontinence by means of a bladder diary functioned as an intervention in itself. Other studies have found bladder diaries to be a way of modifying behavior [34]. In addition, the extra attention given by caregivers may have supported a change in behavior. Thus, it may be more appropriate to consider the control group as a second intervention group that received attention and monitoring of UI. It is also known that UI in older persons is of a transient nature, arising suddenly and being related to reversible causes [35]. In our sample, most participants had not received a diagnosis of UI from a doctor and were unaware of the cause of their involuntary urine loss. It should be pointed out that physical performance was the only outcome measure (primary or secondary) that was measured with an objective test while UI status was evaluated by self-report and the reported results therefor less reliable.

There was no significant effect on the quality of life measures. It may be that a small change in incontinence frequency or physical performance may not necessarily lead to a detectable effect on a general quality of life measure. Most participants had at least one other chronic condition and were dependent on care from others. Nevertheless, even in frail, functionally and cognitively impaired nursing home residents, changes in continence status have a negative impact on quality of life and need to be addressed [36].

Other studies have reported positive effects of PFMT and/or BT in elderly women with a 50%-74% decrease in incontinence episodes in community-dwelling older people, including those who are homebound [12,37]. However, these participants were younger, more highly educated, and more independent than our participants.

Other exercise programs have been proven effective in nursing home residents, and even in older individuals with mental or physical impairment. There was longer endurance, a reduction in incontinence episodes [20], and subjective improvement of urine loss [38]. Unlike ‘Incondition’, these programs use an individualized training schedule with assistance. In these types of program gaining compliance of the nursing staff is problematic [21]. Although group- and individual-based programs are equally effective in younger people [39], it is very possible that disabled older individuals may benefit more from an individual approach. One study that evaluated such an approach in frail elderly women living in Dutch nursing homes, resulted in an improvement in UI status, but one that was not significantly different from the control group [22]. One of the aims in this study was to improve functional capacities by training toileting skills, which was, as in our study, the only significant result. According to the authors one reason for the lack of effect of the intervention, was the lack of motivation of participants, resulting in dropout and reduced power of the study [22]. Nevertheless these and other studies [40] show that in motivated frail elderly, functional improvement is feasible, even with relatively low intensity exercise regimes.

### Limitations of the study

It proved difficult to recruit homes for the study. Some declined because of the anticipated high workload and expected difficulty with adherence to the research protocol, while others were unable to recruit the minimum of 6 participants.

Overall, there was a considerable drop out rate among participants during the study period. For disabled care-dependent women with several chronic conditions a relatively long study period can be too demanding [41]. Those who withdrew had relatively few or no symptoms and were therefore less motivated to complete the study. In younger women the level of severity of UI also influenced adherence to training [42]. However, in the imputation analyses we tried to account for possible selection bias by including all outcome measures and possible confounders described in Table 1 and Table 2 in the model. Unfortunately the imputation program did not take the multilevel structure for dichotomized outcomes into account. Therefore we did not apply multilevel analysis.

Resistance by participants and nursing staff to diagnostic testing meant it was impossible to establish the presence of UI through validated medical tests. This has two important implications. First, we had no information on the type, severity or cause of incontinence among participants. This means that the population studied was probably heterogeneous and may have been more receptive to individualized treatment approaches. Second, it proved impossible to establish the performance of the pelvic floor muscle and bladder function prior to and at the end of the program. Therefore, based on these results no conclusions can be drawn about the role of functional performance in causing or reducing UI.

These limitations are not rare: heterogeneity of the population, lack of standardized terminology, lack of validated instruments, and lack of long-term follow-up are frequently encountered [43]. Moreover, even our relatively simple measurements could not always be carried out as intended.

The frailty of the participants also meant that the training had to be of low intensity, which led to a longer training period which may have increased the drop out rate. This also influenced the adherence to the exercise regimen, which has been shown to be a consistent predictor of responsiveness to behavioral therapy [12].

It was not possible to reach our target of 103 participants per group and therefore enough statistical power. Because of drop out and possible selection bias, it is difficult to generalize our results to the entire population of homes for the elderly. The results from the per protocol analysis therefore only apply to participants, who are motivated, have serious UI symptoms, and do not need assistance with toileting. Over the course of our study, it became apparent that in most homes the emphasis is on controlling incontinence at an institutional level rather than on solving or preventing it at an individual level which may limit future implementation of effective incontinence interventions [44-46]. Staff of the homes, especially caregivers, had difficulty in maintaining the study protocol in participating clients and carrying out measurements. Some of this resistance originated from staff shortages and high workload. This shows that controlled studies in real life settings in which UI care is not a high priority is difficult. Nevertheless, fundamental changes in daily routine are necessary to implement effective interventions in incontinence care [23,24,38]. In the Netherlands improvements have been made in this area including increased diagnostic testing for the cause of incontinence [47].

## Conclusions

In conclusion, our results show it is feasible to improve physical performance in older women in homes for the elderly by a group-based exercise program, but this does not lead to a reduction in UI. These results also show that it is possible to reduce the problem of self-reported UI in women in homes for the elderly. Attention to and monitoring of UI seem already to have led to a decrease in the occurrence of UI and higher priority should be given to the prevention and reduction of UI in this care setting.

## Competing interests

The authors declare that they have no competing interests.

## Authors’ contributions

ET designed the study, recruited and instructed homes, developed and supervised data collection, performed data analysis and drafted the manuscript. AvH developed the intervention and instructed physiotherapists and participating homes. PvD advised and performed part of the statistical analysis (power calculation, regression analysis, imputation). MH participated in designing the study and helped draft the manuscript. All authors read and approved the final manuscript.

## Pre-publication history

The pre-publication history for this paper can be accessed here:

http://www.biomedcentral.com/1471-2318/12/51/prepub
